# Whole Exome Sequencing Suggests Much of Non-BRCA1/BRCA2 Familial Breast Cancer Is Due to Moderate and Low Penetrance Susceptibility Alleles

**DOI:** 10.1371/journal.pone.0055681

**Published:** 2013-02-08

**Authors:** Francisco Javier Gracia-Aznarez, Victoria Fernandez, Guillermo Pita, Paolo Peterlongo, Orlando Dominguez, Miguel de la Hoya, Mercedes Duran, Ana Osorio, Leticia Moreno, Anna Gonzalez-Neira, Juan Manuel Rosa-Rosa, Olga Sinilnikova, Sylvie Mazoyer, John Hopper, Conchi Lazaro, Melissa Southey, Fabrice Odefrey, Siranoush Manoukian, Irene Catucci, Trinidad Caldes, Henry T. Lynch, Florentine S. M. Hilbers, Christi J. van Asperen, Hans F. A. Vasen, David Goldgar, Paolo Radice, Peter Devilee, Javier Benitez

**Affiliations:** 1 Human Genetics Group, Spanish National Cancer Centre (CNIO), Madrid, Spain; 2 Human Genotyping Unit –CEGEN, Spanish National Cancer Centre (CNIO), Madrid, Spain; 3 IFOM, Fondazione Istituto FIRC di Oncologia Molecolare, Milan, Italy; 4 Unit of Molecular Bases of Genetic Risk and Genetic Testing, Department of Preventive and Predictive Medicine, Fondazione IRCCS Istituto Nazionale dei Tumori, Milan, Italy; 5 Genomics Unit, Spanish National Cancer Centre (CNIO), Madrid, Spain; 6 Laboratorio de Oncología Molecular, Instituto de Investigación Sanitaria San Carlos (IdISSC), Madrid, Spain; 7 Instituto de Biología y Genética Molecular (IBGM-UVA.CSIC), Valladolid, Spain; 8 Bioinformatics Unit, Sistemas Genómicos, Valencia, Spain; 9 CNRS UMR5286 INSERM U1052, Université Lyon 1, Cancer Research Center of Lyon, Center Léon Bérard, Lyon, France; 10 Unité Mixte de Génétique Constitutionnelle des Cancers Fréquents, Hospices Civils de Lyon, Centre Léon Bérard, Lyon, France; 11 Centre for Molecular, Environmental, Genetic and Analytical Epidemiology, School of Population Health, The University of Melbourne, Victoria, Australia; 12 Molecular Diagnostic Unit, Hereditary Cancer Program, Institut Català d’Oncologia-IDIBELL, Barcelona, Spain; 13 Genetic Epidemiology Laboratory, Department of Pathology, The University of Melbourne, Victoria, Australia; 14 Unit of Medical Genetics, Department of Preventive and Predictive Medicine, Fondazione IRCCS Istituto Nazionale dei Tumori, Milan, Italy; 15 Department of Preventive Medicine and Public Health, Creighton University School of Medicine, Omaha, Nebraska, United States of America; 16 Department of Human Genetics, Leiden University Medical Center, Leiden, The Netherlands; 17 Department of Clinical Genetics, Leiden University Medical Center, Leiden, The Netherlands; 18 Foundation for the Detection of Hereditary Tumors, Leiden, The Netherlands; 19 Department of Gastroenterology, Leiden University Medical Center, Leiden, The Netherlands; 20 Department of Dermatology, University of Utah School of Medicine, Salt Lake City, Utah, United States of America; 21 Department of Pathology, Leiden University Medical Center, Leiden, The Netherlands; 22 Biomedical Network on Rare Diseases (CIBERER), Madrid, Spain; Ohio State University Medical Center, United States of America

## Abstract

The identification of the two most prevalent susceptibility genes in breast cancer, *BRCA1* and *BRCA2*, was the beginning of a sustained effort to uncover new genes explaining the missing heritability in this disease. Today, additional high, moderate and low penetrance genes have been identified in breast cancer, such as *P53*, *PTEN*, *STK11*, *PALB2* or *ATM*, globally accounting for around 35 percent of the familial cases. In the present study we used massively parallel sequencing to analyze 7 *BRCA1*/*BRCA2* negative families, each having at least 6 affected women with breast cancer (between 6 and 10) diagnosed under the age of 60 across generations. After extensive filtering, Sanger sequencing validation and co-segregation studies, variants were prioritized through either control-population studies, including up to 750 healthy individuals, or case-control assays comprising approximately 5300 samples. As a result, a known moderate susceptibility indel variant (*CHEK2* 1100delC) and a catalogue of 11 rare variants presenting signs of association with breast cancer were identified. All the affected genes are involved in important cellular mechanisms like DNA repair, cell proliferation and survival or cell cycle regulation. This study highlights the need to investigate the role of rare variants in familial cancer development by means of novel high throughput analysis strategies optimized for genetically heterogeneous scenarios. Even considering the intrinsic limitations of exome resequencing studies, our findings support the hypothesis that the majority of non-*BRCA1*/*BRCA2* breast cancer families might be explained by the action of moderate and/or low penetrance susceptibility alleles.

## Introduction

From the original publication of the two most widely known breast cancer (BC) susceptibility genes, namely *BRCA1* and *BRCA2* [1], [2], there has been an active pursuit of new genes contributing to the familial BC phenotype. Additional high penetrance genes have been identified for this disease, such as *P53*, *PTEN* or *STK11* [3], [4], [5], however their number is not yet as high as originally expected. Fanconi Anemia (FA) pathway genes have also been found implicated in BC as moderate penetrance genes, given their incomplete segregation in affected families. Such is the case of *PALB2*, *BRIP1*, *RAD51C* and *XRCC2* [6], [7], [8], [9], which present similar penetrance to other non-FA genes like *ATM*, *CHEK2, RAD51D and RAD51B* [10], [11], [12], [13]. Finally, 21 low risk alleles (odds ratio (OR) between 1.1 and 1.3) identified mainly through GWAS studies [14], [15], [16], [17], [18], [19] complete the picture of known BC susceptibility genes to date. However, all of the variants described so far account for less than 35 percent of familial risk of BC, leaving ample room for uncovering additional germline mutations that confer risk of this disease.

In this regard, linkage analysis has been one of the most widespread techniques for the identification of susceptibility genes. Our group presented one such study in 41 BC families, which revealed three genomic regions of interest [20]. This and other linkage studies have found evidence for more than 20 genomic regions in BC, but none have reached conventional levels of evidence to be validated as true BC susceptibility loci, suggesting a high degree of genetic heterogeneity as well as the need for new approaches to isolate single causal genes.

Massively parallel sequencing was demonstrated to be a good strategy for the identification of genes responsible for monogenic diseases [21], [22], [23] or diseases with a high degree of genetic heterogeneity [24]. The latter would apparently be the case for breast and ovarian cancer syndromes, where each of the identified genes belonging to the FA or other DNA repair pathways explain around 1% of the families with the disease. However, a third option in the form of a polygenic model of inheritance needs to be taken into consideration, especially since this scenario would represent a challenge for the identification of novel susceptibility genes using common massive resequencing strategies. Therefore, careful study design based in either specific phenotypic characteristics or differentially expressed tumoral findings needs to be implemented for efficacy in whole exome sequencing analysis.

In the present study we employed massively parallel sequencing technology to investigate 7 families with at least 6 women (between 6 and 10) bearing unilateral and/or bilateral BC, previously tested negative for *BRCA1/BRCA2* mutations and younger than 60 y/o. Here, we found several rare variants that could putatively act as BC susceptibility genes but we also highlight the lack of evidence of novel high penetrance genes in this disease.

## Materials and Methods

### Ethics Statement

Written informed consent was obtained from all patients included in this study. The research project was approved by the following ethics committees: Instituto de Salud Carlos III Ethics Committee (Spain), Fondazione IRCCS Istituto Nazionale Tumori Ethical Committee (Italy), Human Research Ethics Committee of the University of Melbourne (Australia), Comité Ético de Investigación Clínica del Hospital Clínico San Carlos de Madrid (Spain), Human Research Ethics Committee of Bellvitge University Hospital (Spain), Creighton University Ethics Committee (US), Medical Ethics Committee of the Leiden University Medical Center (The Netherlands).

### Family/sample Selection

Families from five countries (France, Italy, Netherlands, Australia and Spain), including several BC-affected individuals, were evaluated for the present study. Seven families were finally selected based on having at least 6 BC cases (between 6 and 10) diagnosed before the age of 60, being negative for mutations in *BRCA1* and *BRCA2* genes and having no women affected with ovarian cancer in the family. When possible, genomic DNA samples from two individuals per family were selected for whole exome sequencing, while samples from additional individuals in the family were obtained for further validation (Figures S1 and S2).

For case control association studies we selected index cases from 3694 high risk breast/ovarian cancer families without deleterious mutations in *BRCA1* or *BRCA2* (named BRCAx families). Briefly, families contained at least three females affected with breast cancer or at least two first-degree females affected with breast cancer (at least one of them diagnosed before 50) or at least one case of female breast cancer and at least one case of either ovarian, female bilateral breast or male breast cancer. Control population consisted of DNA samples from 3960 women aged between 25 and 65 years and without personal or familial antecedents of any cancer cases. Controls proceeded from the different countries and their distribution is shown in Table S1.

### Exome Capture/massively Parallel Sequencing

DNA samples from eleven cases (Figures S1 and S2) and seven controls (HapMap cell lines Na11881, Na12144, Na12750, Na12761, Na12763, Na12813 and Na12892; Coriell Cell Repositories) were captured and enriched using SureSelect Human All Exon Kit (Agilent Technologies). Enriched samples were sequenced on an Illumina Genome Analyzer II, using two lanes per sample and 78 base-pair, paired-end technology (ArrayExpress accession E-MTAB-1172). Illumina’s Real Time Analysis software version 1.6 (with standard parameters) was used for real-time sequencing image analysis and base calling.

### Next Generation Sequencing Analysis Pipeline

Raw sequencing data for cases and controls was first filtered to remove those reads with (1) no base called in more than 5 positions and (2) having called the same base in more than 70% of the total read length. After filtering, reads were aligned against the human reference genome (hg18) using Novoalign version 2.06.09 (www.novocraft.com) applying standard parameters except for the option of reporting three alignments where multiple alignment sites were found. Those reads not meeting Novoalign’s quality criteria or not matching the reference genome were removed. Samtools version 0.1.8 [25] was then used for PCR duplicate removal as well as for SNP and short INDEL calling in the exomic region. Two scores, namely Depth Score (DS) and Quality Score (QS), were calculated (where possible) as a ratio of the read depth and the Phred Scaled Quality respectively between the variant and reference alleles in a percentage format.

INDELs and heterozygote SNP variants were selected and filtered according to the following criteria:

1. Being common to both sequenced members of the same family (where 2 individuals of the same family were available).
2. Not being present in any of the 7 HapMap controls.
3. Not being present in dbSNP130.

Remaining variants were annotated using Annovar [26] and filtered by functional consequence (e.g. variants identified as intronic, intergenic or synonymous were discarded). Finally, strong candidate variants were obtained after filtering by gene function, focusing on those genes with a potential role in cancer, and score:

- SNPs were filtered by DS and QS, selecting those with a DS and QS between 20 and 210.
- INDELs were filtered by DS <140, Phred Scaled Quality >10, SNP Quality >10 and having at least 3 reads supporting each reference and variant alleles.

In order to set up our score filtering criteria, SNPs and INDELs from the seven HapMap controls already present in dbSNP130 were selected. Several combinations of scores were tested for selecting the highest possible number of variants present in dbSNP130, while targeting the lowest number of total variants detected. For SNPs, the selected combination of scores targeted over 95% of the total number of SNPs detected, while for indels the percentage was reduced to 22% due to the high number of false positive calls in our unfiltered indel data set.

Candidate variants obtained after all the filtering steps were prioritized by Fisher’s Exact Test p value, OR value, segregation and control population studies as described in the text.

### Specificity and Sensitivity in Variant Detection

Sensitivity confirmation analysis for our pipeline, in the form of several modifications to the standard variant filtering strategy, were performed in order to validate the best possible sensitivity for candidate variant detection. These modifications included:

1. Manual re-analysis of all variants in every family after removing variant type and gene filters.
2. Contrasting our set of unfiltered SNP variants against those in 1000 Genomes Project. Common variants presenting minor allele frequencies (MAF) below or equal to 1% were reviewed to guarantee no potential causal variants were missed in the original dbSNP130 filtering step.
3. All stop, frameshift and splicing variants regardless of gene and score filters were re-analysed.
4. Our unfiltered set of variants was matched against some of the latest identified risk modifier variants in BC, while all variants in moderate penetrance genes *XRCC2*, *RAD51C*, *RAD51D*, *PALB2*, *BRIP* and other DNA repair genes were investigated in greater detail.
5. Interesting variants not shared by both individuals in those families with two individuals available for massively parallel sequencing were evaluated. This was conceived as a confirmation test to rule out the possibility of having selected a phenocopy for our initial sequencing analysis in one of the families.

### Variant Validation Studies

All candidate variants were manually matched against the latest available version of Ensembl (www.ensembl.org) for further information and validated by Sanger sequencing.

Segregation studies were performed in additional non-next generation sequenced individuals in each family. Basically, target regions including the variants of interest were amplified by PCR technology using a suitable primer pair and Sanger sequenced. Sequencing results were evaluated with the help of Finch TV trace viewer (Geospiza).

Genotyping in the control collection was done by dHPLC screening or TaqMan (Applied Biosciences). Briefly, appropriate amplification primers were designed for a genomic region containing the variant of interest. Then, genomic control DNA from individuals without familial BC antecedents was PCR amplified and analysed by dHPLC as previously described [27]. DNAs corresponding to chromatography patterns departing from that of an internal control (which does not present mutations in the fragment of interest), were further studied by Sanger sequencing. In those cases where TaqMan was the technique of choice, custom probes were ordered directly from Applied Biosciences and used according to standard protocol. Results were obtained on a 7900HT Sequence Detection System (Applied Biosciences) and analyzed using SDS 2.4 allelic discrimination software.

Case control studies were performed either using TaqMan genotyping assays or TaqMan OpenArray technology (Applied Biosciences). Genotyping using TaqMan assays was performed as previously described, this time including both familial BC affected (cases) and non-affected (control) individuals. TaqMan OpenArray technology was used for large scale genotyping. Briefly, 64 SNP array format was selected to genotype 56 variants of unknown significance in 2693 cases and 2544 controls from different ethnic origin coming from various centers from the BC consortium. Issues with custom probe design and cluster discrimination in specific probes reduced the final number of informative variants to 39. A total of 130 arrays were performed and analyzed uniformly according to the manufacturer’s standard protocol. HapMap and sample duplicates were included in each array to serve as internal controls and to ensure reproducibility of the results. Genotype calling and sample clustering for OpenArray assays was performed in TaqMan Genotyper Software v1.0 (Applied Biosciences). Statistical analysis of the data was done using PLINK software [28], where Fisher’s Exact Test p-values, OR and 95% confidence intervals for the OR were computed.

### Predictive Programs

The potential functional impact of the non-synonymous variants detected in this study was inferred based on predictions made by SIFT [29] and PolyPhen [30]. Pathogenicity of variants that were located in intronic positions that could potentially affect the splicing process was predicted based in the algorithms integrated in the software Alamut 2.0 (SpliceSiteFinder-like, MaxEntScan, NNSPLICE and Human Splicing Finder).

## Results

Eleven individuals from 7 families and 7 HapMap cell lines, serving as internal and filtering controls, were analyzed by massively parallel sequencing in this study. Results including number of reads, sample coverage and sequencing depth have been summarized in Table S2.

**Figure 1:**
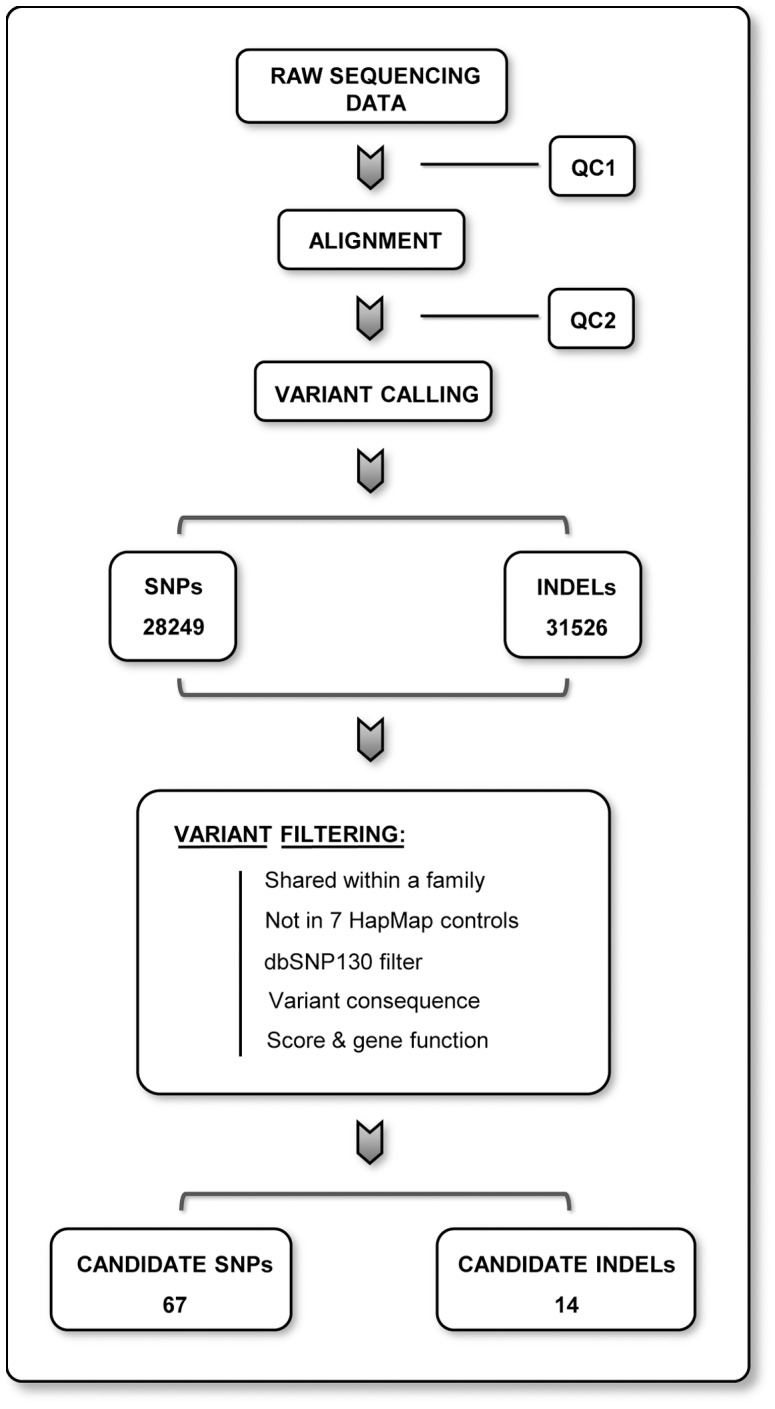
Summary of the data analysis pipeline followed in the present study. Raw sequencing data was screened for common artifacts prior to the alignment step in a first quality control phase (QC1). High quality (QC2) genome matches were analyzed for variants, in the form of departures from a consensus reference genome. Subsequently, variants were filtered by keeping those common to both members in each family and then discarding variants present in HapMap controls and dbSNP130. Further filtering by variant consequence, score and gene function (see material and methods for details) resulted in a list of 67 snps and 14 indel candidate variants.

The bioinformatics analysis and variant filtering pipeline was developed by our group and recently used in the identification of a novel familial pheocromocytoma gene [24]. The main steps are represented in Figure 1. After applying this filtering pipeline, an initial set of 67 (average of 10 per family) SNPs and 14 INDELs (average of 2 per family) passed all the filters (Tables 1 and 2).

**Table 1:** SNP Filtering Summary.

Sample	DetectedheterozygousSNPs	Commonwithin a family	Not present in 7 HapMap Controls	Not present in dbSNP130	After variant consequencefilter^a^	After score and genefunction filters^b^
07S240 (Pedigree 49)	29549	29549	9451	3838	1000	67^d^
DAD_1 (Pedigree 694)	28697	28697	9144	3666	899	
F2887_13 (Pedigree2887)	28978	9271^c^	1681^c^	404^c^	156^c^	
F2887_24 (Pedigree2887)	30691					
F3311_5 (Pedigree3311)	26774	8776^c^	1686^c^	328^c^	100^c^	
F3311_43 (Pedigree3311)	26468					
I_1408 (Pedigree 531)	28841	28841	9022	3553	971	
RUL036_2 (Pedigree RUL036)	27060	11405^c^	2746^c^	776^c^	188^c^	
RUL036_7 (Pedigree RUL036)	27032					
RUL153_2 (Pedigree RUL153)	26908	8295^c^	1373^c^	197^c^	53^c^	
RUL153_3 (Pedigree RUL153)	27406					
**Average**	**28249**	**17833**	**5015**	**1823**	**481**	**10**
**Percentage remaining**	**100**	**63.13**	**17.75**	**6.45**	**1.70**	**0.04**

Variant filtering representation through the number of SNPs remaining after the various filtering steps.

The Average and Percentage remaining rows represent the average number of variants and percentage of variants remaining per family.

a Intronic, intergenic and synonymous variants were discarded. See methods.

b Detailed criteria for these filters is reported on the methods section.

c Number of variants shared between the two individuals in the family.

d Total number of final variants for all the individuals in this study.

**Table 2:** INDEL Filtering Summary.

Sample	Detected INDELs	Common within a family	Not present in 7 HapMap Controls	Not present in dbSNP130	After variant consequence filter^a^	After score and gene function filters^b^
07S240 (Pedigree 49)	36189	36189	26077	24580	11387	14^d^
DAD_1 (Pedigree 694)	35606	35606	25741	24204	11046	
F2887_13 (Pedigree2887)	34081	12299^c^	5314^c^	4650^c^	1354^c^	
F2887_24 (Pedigree2887)	31445					
F3311_5 (Pedigree3311)	30983	12442^c^	4982^c^	4340^c^	579^c^	
F3311_43 (Pedigree3311)	26441					
I_1408 (Pedigree 531)	36131	36131	26082	24591	11352	
RUL036_2 (Pedigree RUL036)	25162	13042^c^	5087^c^	4398^c^	587^c^	
RUL036_7 (Pedigree RUL036)	25652					
RUL153_2 (Pedigree RUL153)	25698	11878^c^	4445^c^	3834^c^	506^c^	
RUL153_3 (Pedigree RUL153)	26045					
**Average**	**31526**	**22512**	**13961**	**12942**	**5259**	**2**
**Percentage remaining**	**100**	**71.41**	**44.28**	**41.05**	**16.68**	**0.01**

Variant filtering representation through the number of INDELs remaining after the various filtering steps.

The Average and Percentage remaining rows represent the average number of variants and percentage of variants remaining per family.

a Intronic and intergenic variants were discarded. See methods.

b Detailed criteria for these filters is reported on the methods section.

c Number of variants shared between the two individuals in the family.

d Total number of final variants for all the individuals in this study.

First, we focused on a group of 25 high interest candidate variants including 12 indels, 3 stopgains, and 6 splicing variants. Additionally, 4 non-synonymous SNPs were included in this group because they either were present in two families (*FANCM*, *CNTROB*) or affected a *P53*-related gene, *TP53I13* (Table S3). Validation of the final candidates was done by Sanger sequencing and segregation. Then, we performed segregation studies in other affected members of the same family from whom DNA was available (an average of 2–4 additional members/family except in family 694). Furthermore, those variants segregating in over 50% of the available individuals in each family were tested in a pool of up to 750 Spanish control women.

One of the variants identified in Family RUL153 was a deletion in *CHEK2* (c.1100delC), a gene already described as a moderated susceptibility gene [11]. We extended the study to two additional affected members in this family and found that one of them (I-1) didn’t present the same mutation (Figure S1). The non-carrier showed an elevated diagnostic age (76 years old) when compared to the rest of the affected carrier individuals, so a haplotype analysis was performed and helped to confirm this individual as a phenocopy (data not shown).

*FANCM* was mutated in families 694 and 531 (Figure S1). The former family carried the p.Arg1931 stop mutation (c.5791C>T) annotated as rs144567652 while the latter carried a non-synonymous mutation (c.4392A>T), both of them validated by Sanger sequencing. However, a segregation analysis discarded the variant in family 531 as a plausible BC related allele (Table S3). We then centered our study on the stopgain variant in *FANCM*, since some genes from the Fanconi pathway have already been described as BC susceptibility genes [31]. Given that segregation studies were not feasible in this family, in order to analyze the possible effect of this variant we performed a case control association study by TaqMan, including 3409 BRCAX cases and 3896 controls from Italy, Netherlands, Australia and Spain (Table S1). We found 10 positive cases and 5 positive controls, with an estimated OR = 2.29 (95%CI = 0.71–8.54), p = 0.13.

Other candidate variants segregating in more that 50% of the individuals in our families such as *SOSTDC1* (c.664delA) (family 49, involved in cellular proliferation, differentiation, and programmed cell death); *CNTROB* (c.2081A>G, rs139292572) (family 3311, a centrosomal *BRCA2* interacting protein) and *HSP90B1* (c.2362_2364delGAA, rs5800607) (family 3311, codifying for a heat shock protein), were discarded for being present in control population with a MAF >1% (Table S3). On the other hand, *SLBP* (c.697G>A) (a stem-loop binding protein) and *CNTROB* (c.1819G>A), both in family RUL036, were not found in the same set of controls. A splice site variant in the oncogenesis-involved *WNT8A* (c.103G>T) gene was found in family 694. We were unable to confirm experimentally whether the variant altered the splicing, due to a very low expression level of the gene in fibroblasts (data not shown). However, after performing a case-control study by TaqMan in 2043 BRCAx cases and 2186 controls, none of the 4229 samples were found to carry the same variant. Ultimately, four rare variants were selected from the group of 25 high interest variants: FANCM (c.5791C>T, stopgain), SLBP (c.697G>A, splicing), CNTROB (c.1819G>A, missense) and WNT8A (c.103G>T, splicing).

We then focused on the analysis of the remaining 56 missense variants of unknown significance, not previously described in ensemble database and predicted to be deleterious or probably damaging in at least one of the two predictive programs, SIFT [29] and Polyphen [30]. In order to evaluate their role in cancer susceptibility, the variants were genotyped in 2693 BRCAx cases and 2544 controls using an Open Array platform (Applied Biosystems). Technical problems during custom design, as well as discrimination issues in the analysis of specific probes reduced the number of variants finally evaluated to 39. Table S4 includes genotyping-related information on genes, frequencies, OR and p values.

From the previous group of 39 variants, we selected for further validation those variants with an estimated OR higher than two or not present in controls. Thirteen variants fulfilling these criteria were Sanger-validated and analyzed for segregation in their original families (except for variants in family 694) (Table S5). Five variants (in addition to two from family 694) were validated and showed segregation in at least 50% of the available individuals. We consider these seven variants as well as the previous four candidates our potential rare variants candidate to play a role in familial BC development (Table 3).

**Table 3:** Final Candidate Variants.

Pedigree	Chr^a^	Position^b^	Consequence^c^	Alleles Reference/Variant	Gene	Description	Allele frequency cases	Allele frequency controls	P value^d^	OR^e^	95% CI^f^
**694**	14	44737671	Stopgain SNV	C/T	FANCM	Fanconi anemia, complementation groupM [Source:HGNC Symbol;Acc:23168]	0.00147	0.00077	0.13	2.29	0.71–8.54
	5	137448086	exonic; splicing	G/T	WNT8A	wingless-type MMTV integration site family, member 8A [Source:HGNC Symbol;Acc:12788]	0	0	N/A	N/A	N/A
	9	127361802	NS	G/A	MAPKAP1	mitogen-activated protein kinase associated protein 1 [Source:HGNC Symbol;Acc:18752]	0.004278	0.001966	0.03709	2.181	1.037–4.587
	9	116707958	NS	C/T	TNFSF8	tumor necrosis factor (ligand) superfamily, member 8provided by HGNC	0	0	N/A	N/A	N/A
**531**	1	43858431	NS	C/A	PTPRF	protein tyrosine phosphatase, receptor type,F [Source:HGNC Symbol;Acc:9670]	0.00186	0.0007877	0.1818	2.364	0.7409–7.542
	3	69199787	NS	C/T	UBA3	ubiquitin-like modifier activating enzyme 3 [Source:HGNC Symbol;Acc:12470]	0.000186	0	1	N/A	N/A
**RUL036**	16	278080	UTR3	G/A	AXIN1	axin 1 [Source:HGNC Symbol;Acc:903]	0.0007443	0.0001967	0.3758	3.786	0.423–33.89
	22	31584009	NS	G/A	TIMP3	TIMP metallopeptidase inhibitor 3 [Source:HGNC Symbol;Acc:11822]	0.0001859	0	1	N/A	N/A
	4	1665238	exonic; splicing	G/A	SLBP	stem-loop binding protein [Source:HGNC Symbol;Acc:10904]	N/A	0	N/A	N/A	N/A
	17	7789855	NS	G/A	CNTROB	centrobin, centrosomal BRCA2 interacting protein [Source:HGNC Symbol;Acc:29616]	N/A	0	N/A	N/A	N/A
**49**	9	90806008	NS	G/A	S1PR3	sphingosine-1-phosphate receptor 3 [Source:HGNC Symbol;Acc:3167]	0.000186	0	1	N/A	N/A

List of final candidate variants passing all filters.

a Chromosome in which the variant was mapped.

b Position according to the coordinate system (HG18).

c Variant consequence: NS = non-synonymous variant UTR3 = 3′ untranslated region variant.

d Fisher’s Exact Test P value.

e Odds Ratio.

f 95% confidence interval for the Odds Ratio.

N/A = not available.

## Discussion

The present study was designed to identify new high susceptibility genes in familial BC. Family selection was based in the highest possible number BC cases per family under the age of 60 (6 to 10 cases through generations), while having no other tumor types segregating in the family. As proposing candidates relying on next generation sequencing results alone is not sufficient evidence, segregation, control population and case-control studies were performed to gain further insight on the relevance of the proposed candidates in BC.

A total number of 81 variants, 67 snps and 14 indels, were identified as potential candidates after applying our filtering protocols. These were subdivided into two groups to facilitate the analysis, a first group containing mainly putative protein truncating variants or variants shared between several families and a second group of rare missense mutations. Among those in the first group, we detected a previously identified moderate susceptibility indel variant in CHEK2 (c.1100delC) in family RUL153 (Figure S1), which served to confirm the adequacy of our filtering strategy for the detection of known BC-related variants.

Additional variants were also analyzed in this first group. *FANCM* variant c.5791C>T generates a premature stop codon, originating the loss of 118 aminoacids from the c-terminal end of the main transcript and putatively influencing *FANCD2* monoubiquitination. A case control study in a cohort of over 7300 samples revealed an OR = 2.29 and a non-significant Fisher’s Exact Test p value (p = 0.13). However, given the low frequency of this variant (0.0011 in cases and 0.00077 in controls), its variable prevalence in the available populations (0.6% in the Italian, 0.3% in the Spanish, but not found in the Netherland samples and found once (0.14%) in the Australian controls, data not shown) and FANCM’s important role in DNA repair, it would be interesting to analyze this gene in a higher number of samples to fully understand its contribution to BC. Also, hints of a possible link with BC were found for *BRCA2* interacting *CNTROB* (c.1819G>A), *SLBP* (c.697G>A) and *WNT8A* (c.103G>T). Those presented a high level of familiar segregation in the available individuals (greater than 50%) and absence of the variant in over 700 controls (*CNTROB* and *SLBP*) or in over 4000 samples (*WNT8A*), similarly to what could be expected for rare susceptibility variants (Table S5).

To explore the role of the 56 identified missense mutations, we performed a case-control association study in over 5200 samples (BRCAx cases and controls), a large enough cohort to obtain a preliminary idea of association with BC. Since we discarded variants with a MAF higher than 1% and only rare variants were considered after this point in the analysis, we did not have enough statistical power or we could not calculate it in those variants present in neither cases nor controls. Therefore, we selected variants with a high probability of being linked with cancer by prioritizing variants with OR higher than 2 or absent in controls, a damaging predicted functional impact and segregating in over 50% of the available individuals in each family. Our final candidates include 7 variants in genes related with cell survival, proliferation, angiogenesis, cell cycle progression, cell adhesion and other roles plausibly related to cancer development and progression (Table 3). These genes are candidates to be studied by targeted resequencing in a large series of cases (BRCAx) and controls in order to assess their role in familial cancer susceptibility. In this regard, based on the current evidence, we cannot conclude that these variants are pathogenic and they should not be added to mutation databases as pathogenic variants based solely on the data presented here.

As our families were selected for the high number of affected women across generations and their seemingly dominant model of inheritance, our results were unexpected. However, two recent whole exome studies were also unsuccessful in finding novel high penetrance genes in BC, pointing our result towards a more common scenario in this disease. While selecting their families with less strict criteria, Snape et al [32] sequenced 50 BRCAx probands to find no high susceptibility genes. More recently, Thompson et al [33] found two new genes by whole exome sequencing, *FANCC* and *BLM*, which would be mutated in approximately 0.5% of BRCAx families. Both studies also suggested a number of rare variants to be analyzed further in future studies.

A number of limitations in the present study need to be taken into consideration. The number of individuals/families selected for this re-sequencing study is smaller than that of similar studies. While this approach might limit the number of potential candidate variants detected as compared to other less stringent family selection strategies [32], it is a reflection of the small number of non-BRCA1/2 families presenting a high number (from 6 to 10) of BC events per family, which in turn represents a highly desirable starting point for the identification of novel high penetrance genes in any disease. Additionally, the observed phenotype in our families could be related to undetectable alterations using the current approach: mutations in regions outside the captured genomic pool, undetected alterations due to alignment/variant calling errors or those presenting insufficient evidence to pass the filtering criteria. When possible, this and other potential pitfalls in this study, such as rejecting mutations for being in less well known regions (e.g. introns), discarding causal variants for being present in the databases or choosing a phenocopy for the initial re-sequencing in one of the families, were taken into consideration. In this regard, the filtering strategy was conceived to prioritize sensitivity while using filtering principles widely applied in successful massively parallel sequencing studies [24], [32], [34], [35]. The fact that low stringency filtering thresholds paired to the use of alternative analysis to assess the efficacy of most of our filtering steps (see methods) yielded no additional potential candidates further supports our results.

Finally we cannot rule out that some of these families shift a putative explanation towards a polygenic model where moderate and low penetrance alleles would play a predominant role. This hypothesis has been suggested by different authors [36], [37] mainly based in linkage analysis, modeling or targeted resequencing [38] due to the lack of success in finding high susceptibility genes even in regions with statistically significant lod scores. Nowadays, new technologies like massively parallel sequencing in stringently selected families, as in our case, support it from a practical point of view. An example of this is a recent paper by Sawyer et al [39] showing how women affected by familial BC had a highly significant excess of risk alleles compared with controls. Unfortunately, due to the small number of cases in our study we could not analyze this issue.

According to the presented data, families fulfilling the criteria of high risk for BC and including several affected women across generations show evidence of a high genetic heterogeneity and complexity. In this context, some families would be explained based in a polygenic model where rare variants would play an interesting role in BC susceptibility.

Further studies in a larger number of families, using a combined strategy of whole exome/whole genome sequencing, case-control association studies including thousands of samples and targeted resequencing of genomic regions containing rare variants is needed to confirm these preliminary results and to define the genetic architecture of non-BRCA1/2 BC families.

## Supporting Information

Figure S1 **Pedigrees of families included in this study I.** Pedigrees 694, RUL153 and 531 are represented in this figure. Arrows indicate individuals selected for massively parallel sequencing. Cancer affected individuals are partly or totally highlighted in black. Br sign followed by a number under affected individuals marks the age of diagnostics of each breast cancer event. Deceased individuals are marked by a slash. Arabic numbers refer to those members used for segregation. UL = Unilateral breast cancer. BL = Bilateral breast cancer.
(TIF)

Figure S2 **Pedigrees of families included in this study II.** Pedigrees 3311, RUL036, 49 and 2887 are represented in this figure. Arrows indicate individuals selected for next generation sequencing. Cancer affected individuals are partly or totally highlighted in black. Br sign followed by a number under affected individuals marks the age of diagnostics of each breast cancer event. Deceased individuals are marked by a slash. Arabic numbers refer to those members used for segregation. UL = Unilateral breast cancer. BL = Bilateral breast cancer.
(TIF)

Table S1 **Clustering of Validation Samples by Country of Origin.** Distribution of samples and controls used for the different case-control studies and validation analysis by TaqMan and OpenArray. FANCM was studied with samples from 1–6. WNT8A was studied with samples 1, 2, 4 and 5. OpenArray: samples from 1–5. CNIO control samples were used for validation of variants in Table S3. ^a^Spanish National Cancer Research Centre (Spain). ^b^Hospital Clinico San Carlos (Spain). ^c^Hospital Universitario Valladolid (Spain). ^d^Leiden University Medical Center (Netherlands). ^e^Istituto Nazionale dei Tumori (Italy). ^d^The University of Melbourne (Australia).
(DOC)

Table S2 **Next Generation Sequencing Metrics and Coverage.** Next generation sequencing results per sample. ^a^Sequence stands for the number of bases sequenced per individual. ^b^Number of genomic matches refers to the number of genomic locations aligning to the total number of reads in a given sample. The option of reporting up to 3 alignment positions on conflicting matches increases this number with regard to the total number of reads. ^c^On-target refers to the total number of reads on target. ^d^Paired end column represents the number of on-target positions successfully aligned as paired-end reads.
(DOC)

Table S3 **Candidate Variants I: Potential Protein Truncating, Splicing Variants and Indels.** List of 25 potential protein truncating or splicing variants, indels and variants of unknown significance in genes shared by different families. In bold, selected variants. ^a^Position according to the coordinate system (HG18). ^b^Sanger sequencing validation. ^c^Results of segregation studies marking positive individuals out of the total number available for validation. ^d^Control population studies were performed in variants segregating in over 50% of the available individuals for validation. Figures represent the number of positive cases out of the total number tested, as well as the percentage of positive cases in the population. ^e^Minor Allele Frequency reported in 1000 Genomes Project (May 2011 release). N/A = not available. NV = non validated.
(DOC)

Table S4 **List of Variants Analyzed by Open Array Case-control Design and Results.** List of variants analyzed by Open Array case-control design. In bold, selected candidates. ^a^Chromosome where the variant was mapped. ^b^Position according to the coordinate system (HG18). ^c^Variant consequence: NS = non-synonymous variant UTR3 = 3′ untranslated region variant. ^d^Fisher’s Exact Test P value. ^e^Odds Ratio. ^f^95% confidence interval for the Odds Ratio. ^g^Minor Allele Frequency reported in 1000 Genomes Project (May 2011 release). N/A = not available.
(DOC)

Table S5 **Candidate Variants II.** Open Array Variants with an OR higher than 2 or absent in control population. In bold, selected candidates. ^a^Position according to the coordinate system (HG18). ^b^Variant consequence. UTR3 = variant is located at the 3′ untranslated region. NS = non-synonymous variant. ^c^Sanger sequencing validation. ^d^Results of segregation studies stating the number of positive individuals out of the total number available for validation in the family. ^e^SHIFT/Polyphen predictions: D = Damaging PD = Probably damaging T = Tolerated.
(DOC)
