# Supplementary figures and images for: Whole Exome Sequencing Suggests Much of Non-BRCA1/BRCA2 Familial Breast Cancer Is Due to Moderate and Low Penetrance Susceptibility Alleles
Source: PLoS One. 2013 Feb 8;8(2):e55681. doi: 10.1371/journal.pone.0055681 (PMC3568132; doi:10.1371/journal.pone.0055681)

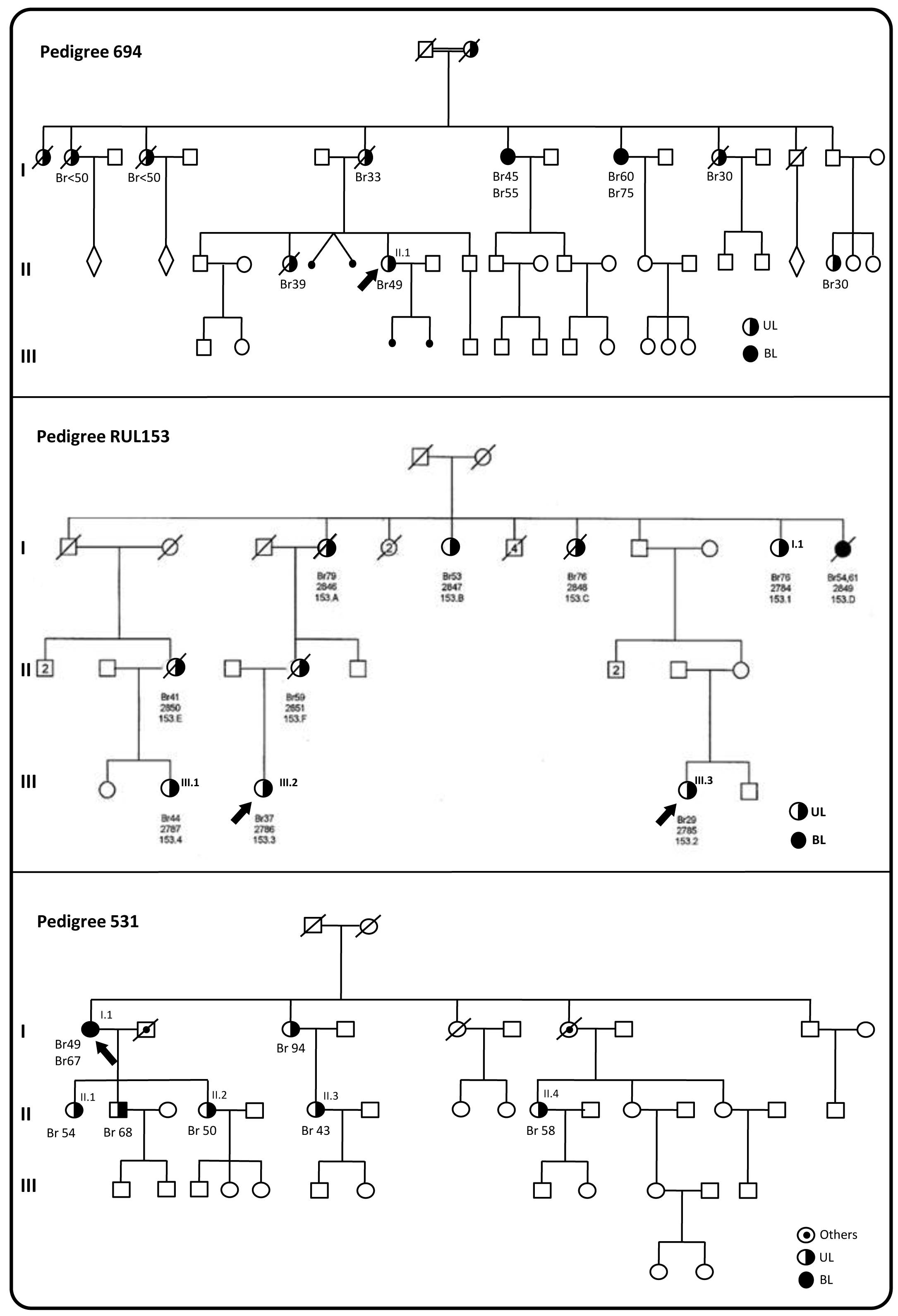

Supplement: Figure S1 — Pedigrees of families included in this study I. Pedigrees 694, RUL153 and 531 are represented in this figure. Arrows indicate individuals selected for massively parallel sequencing. Cancer affected individuals are partly or totally highlighted in black. Br sign followed by a number under affected individuals marks the age of diagnostics of each breast cancer event. Deceased individuals are marked by a slash. Arabic numbers refer to those members used for segregation. UL = Unilateral breast cancer. BL = Bilateral breast cancer. (TIF) [file pone.0055681.s001.tif]

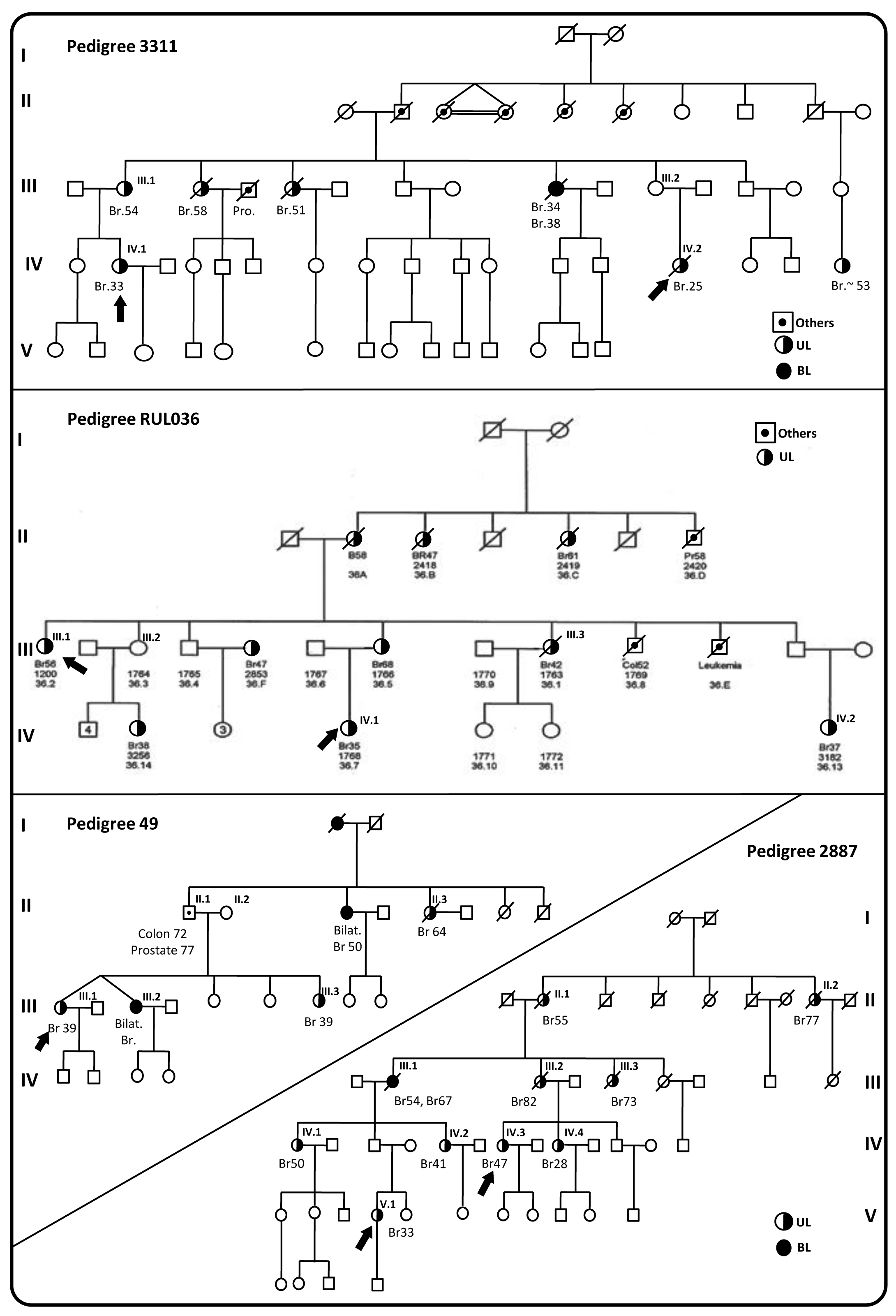

Supplement: Figure S2 — Pedigrees of families included in this study II. Pedigrees 3311, RUL036, 49 and 2887 are represented in this figure. Arrows indicate individuals selected for next generation sequencing. Cancer affected individuals are partly or totally highlighted in black. Br sign followed by a number under affected individuals marks the age of diagnostics of each breast cancer event. Deceased individuals are marked by a slash. Arabic numbers refer to those members used for segregation. UL = Unilateral breast cancer. BL = Bilateral breast cancer. (TIF) [file pone.0055681.s002.tif]
