# Supplementary material for: Whole Exome Sequencing Suggests Much of Non-BRCA1/BRCA2 Familial Breast Cancer Is Due to Moderate and Low Penetrance Susceptibility Alleles
Source: PLoS One. 2013 Feb 8;8(2):e55681. doi: 10.1371/journal.pone.0055681 (PMC3568132; doi:10.1371/journal.pone.0055681)
Supplement: Table S1 — Clustering of Validation Samples by Country of Origin. Distribution of samples and controls used for the different case-control studies and validation analysis by TaqMan and OpenArray. FANCM was studied with samples from 1–6. WNT8A was studied with samples 1, 2, 4 and 5. OpenArray: samples from 1–5. CNIO control samples were used for validation of variants in Table S3. aSpanish National Cancer Research Centre (Spain). bHospital Clinico San Carlos (Spain). cHospital Universitario Valladolid (Spain). dLeiden University Medical Center (Netherlands). eIstituto Nazionale dei Tumori (Italy). dThe University of Melbourne (Australia). (DOC) [file pone.0055681.s003.doc]

**Table S1. Clustering of Validation Samples by Country of Origin.**

|  | **CNIO (ES-1)^a^** | **HCSC (ES-2) ^b^** | **HUV (ES-3)^c^** | **NTL(4)^d^** | **IT(5)^e^** | **AUS(6)^f^** | **TOTAL** |
| --- | --- | --- | --- | --- | --- | --- | --- |
| CASES | 752 | 316 | 727 | 463 | 1030 | 406 | 3694 |
| CONTROLS | 1035 | 187 | 0 | 419 | 1618 | 701 | 3960 |

Distribution of samples and controls used for the different case-control studies and validation analysis by TaqMan and OpenArray.

FANCM was studied with samples from 1-6. WNT8A was studied with samples 1, 2, 4 and 5. OpenArray: samples from 1-5. CNIO control samples were used for validation of variants in Table S3.

^a^ Spanish National Cancer Research Centre (Spain)

^b^ Hospital Clinico San Carlos (Spain).

^c^ Hospital Universitario Valladolid (Spain).

^d^ Leiden University Medical Center (Netherlands).

^e^ Istituto Nazionale dei Tumori (Italy).

^d^ The University of Melbourne (Australia).
