# Supplementary material for: Whole Exome Sequencing Suggests Much of Non-BRCA1/BRCA2 Familial Breast Cancer Is Due to Moderate and Low Penetrance Susceptibility Alleles
Source: PLoS One. 2013 Feb 8;8(2):e55681. doi: 10.1371/journal.pone.0055681 (PMC3568132; doi:10.1371/journal.pone.0055681)
Supplement: Table S2 — Next Generation Sequencing Metrics and Coverage. Next generation sequencing results per sample. aSequence stands for the number of bases sequenced per individual. bNumber of genomic matches refers to the number of genomic locations aligning to the total number of reads in a given sample. The option of reporting up to 3 alignment positions on conflicting matches increases this number with regard to the total number of reads. cOn-target refers to the total number of reads on target. dPaired end column represents the number of on-target positions successfully aligned as paired-end reads. (DOC) [file pone.0055681.s004.doc]

**Table S2. Next Generation Sequencing Metrics and Coverage.**

|  | **Sample** | **Number of reads** | **Sequence^a^** | **Number of genomic matches^b^** | **On-target +/-200bp (%)** | **On-target (%)^c^** | **On-target paired-end (%)^d^** | **Coverage ≥1x (% of target regions)** | **Coverage ≥20x (% of target regions)** | | **Mean read depth** | **Median read depth** | **Regions 5 ≤ read depth ≤ 215 (%)** |
| --- | --- | --- | --- | --- | --- | --- | --- | --- | --- | --- | --- | --- | --- |
| **Controls** | NA11881 | 122354518 | 9543652404 | 135879100 | 82132028 (60.44%) | 75893477 (55.85%) | 74844641 (98.62%) | 37398337 (99.36%) | 33725985 (89.60%) | 135.06 | | 109 | 29487802 (78.34%) |
|  | NA12144 | 139515778 | 10882230684 | 153936627 | 116158981 (75.46%) | 99838815 (64.86%) | 98362540 (98.52%) | 37424328 (99.43%) | 33715462 (89.57%) | 135.58 | | 109 | 29486999 (78.34%) |
|  | NA12750 | 125830680 | 9814793040 | 140060439 | 92690558 (66.18%) | 81186046 (57.97%) | 79013978 (97.32%) | 37422109 (99.42%) | 33131638 (88.02%) | 116.98 | | 95 | 31513590 (83.72%) |
|  | NA12761 | 102933348 | 8028801144 | 113394946 | 61077903 (53.86%) | 56073434 (49.45%) | 55419317 (98.83%) | 37403783 (99.37%) | 33524765 (89.07%) | 130.50 | | 105 | 29922847 (79.50%) |
|  | NA12763 | 105910626 | 8261028828 | 112547964 | 55740245 (49.53%) | 50687853 (45.04%) | 50191922 (99.02%) | 37366823 (99.27%) | 33507838 (89.02%) | 134.60 | | 109 | 29384087 (78.07%) |
|  | NA12813 | 118799624 | 9266370672 | 131565041 | 83180907 (63.22%) | 76553941 (58.19%) | 75712836 (98.90%) | 37386973 (99.33%) | 33572265 (89.19%) | 136.26 | | 109 | 29270432 (77.76%) |
|  | NA12892 | 111710036 | 8713382808 | 124123147 | 73409558 (59.14%) | 67609662 (54.47%) | 66754505 (98.76%) | 37372548 (99.29%) | 33276872 (88.41%) | 128.48 | | 102 | 30114170 (80.00%) |
| **Cases** | 07S240 (Family 49) | 124702314 | 9726780492 | 136885211 | 93140180 (68.04%) | 79935515 (58.40%) | 79364100 (99.29%) | 37374136 (99.29%) | 32674941 (86.81%) | 108.33 | | 85 | 32047920 (85.14%) |
|  | DAD_1 (Family 694) | 130181634 | 10154167452 | 143487243 | 98063187 (68.34%) | 83632734 (58.29%) | 83150967 (99.42%) | 37485794 (99.59%) | 34374298 (91.32%) | 100.74 | | 75 | 33411872 (88.77%) |
|  | F2887_13 (Family 2887) | 128117542 | 9993168276 | 140915720 | 97409560 (69.12%) | 83825661 (59.49%) | 83392089 (99.48%) | 37191535 (98.81%) | 30092381 (79.95%) | 70.41 | | 59 | 34835926 (92.55%) |
|  | F2887_24 (Family 2887) | 115682414 | 9023228292 | 127522927 | 88625162 (69.50%) | 76948655 (60.34%) | 76535164 (99.46%) | 37111019 (98.59%) | 30810133 (81.85%) | 79.41 | | 64 | 33937374 (90.16%) |
|  | F3311_5 (Family 3311) | 135500012 | 10569000936 | 149218406 | 112993679 (75.72%) | 95952265 (64.30%) | 95354040 (99.38%) | 37140789 (98.67%) | 28122880 (74.71%) | 75.17 | | 54 | 33186504 (88.17%) |
|  | F3311_43 (Family 3311) | 140545388 | 10962540264 | 154899341 | 114457573 (73.89%) | 97484593 (62.93%) | 96856784 (99.36%) | 36899738 (98.03%) | 30664734 (81.47%) | 88.02 | | 69 | 32986722 (87.64%) |
|  | I_1408 (Family 531) | 127690794 | 9959881932 | 140184885 | 96834105 (69.08%) | 83579494 (59.62%) | 82918852 (99.21%) | 37162205 (98.73%) | 31944432 (84.87%) | 88.86 | | 73 | 33752835 (89.67%) |
|  | RUL036_2 (Family RUL036) | 134540338 | 10494146364 | 148056828 | 108993122 (73.62%) | 92824432 (62.70%) | 92113085 (99.23%) | 37421130 (99.42%) | 32984208 (87.63%) | 112.72 | | 92 | 31934238 (84.84%) |
|  | RUL036_7 (Family RUL036) | 134852704 | 10518510912 | 148675204 | 111866891 (75.24%) | 96300607 (64.77%) | 95445543 (99.11%) | 37410238 (99.39%) | 33017719 (87.72%) | 116.48 | | 94 | 31480265 (83.63%) |
|  | RUL153_2 (Family RUL153) | 134485496 | 10489868688 | 148226089 | 112158192 (75.67%) | 95987483 (64.76%) | 95214543 (99.19%) | 37475702 (99.56%) | 33467683 (88.91%) | 119.08 | | 96 | 31454944 (83.57%) |
|  | RUL153_3 (Family RUL153) | 135943720 | 10603610160 | 150655678 | 112227091 (74.49%) | 94307107 (62.60%) | 93485182 (99.13%) | 37498085 (99.62%) | 33513657 (89.04%) | 109.83 | | 89 | 32637920 (86.71%) |
| **Average** | | **126072054** | **9833620186** | **138901933** | **95064385 (68.44%)** | **82701210 (59.54%)** | **81896116 (99.01%)** | **37330293 (99.18%)** | **32562327 (86.51%)** | **110.36** | | **88.22** | **31713692 (84.25%)** |

Next generation sequencing results per sample.

^a^ Sequence stands for the number of bases sequenced per individual.

^b^ Number of genomic matches refers to the number of genomic locations aligning to the total number of reads in a given sample. The option of reporting up to 3 alignment positions on conflicting matches increases this number with regard to the total number of reads.

^c^ On-target refers to the total number of reads on target.

^d^ Paired end column represents the number of on-target positions successfully aligned as paired-end reads.
