# Supplementary material for: Whole Exome Sequencing Suggests Much of Non-BRCA1/BRCA2 Familial Breast Cancer Is Due to Moderate and Low Penetrance Susceptibility Alleles
Source: PLoS One. 2013 Feb 8;8(2):e55681. doi: 10.1371/journal.pone.0055681 (PMC3568132; doi:10.1371/journal.pone.0055681)
Supplement: Table S3 — Candidate Variants I: Potential Protein Truncating, Splicing Variants and Indels. List of 25 potential protein truncating or splicing variants, indels and variants of unknown significance in genes shared by different families. In bold, selected variants. aPosition according to the coordinate system (HG18). bSanger sequencing validation. cResults of segregation studies marking positive individuals out of the total number available for validation. dControl population studies were performed in variants segregating in over 50% of the available individuals for validation. Figures represent the number of positive cases out of the total number tested, as well as the percentage of positive cases in the population. eMinor Allele Frequency reported in 1000 Genomes Project (May 2011 release). N/A = not available. NV = non validated. (DOC) [file pone.0055681.s005.doc]

**Table S3. Candidate Variants I: Potential Protein Truncating, Splicing Variants and Indels.**

| **Family** | **Chromosome** | **Position^a^** | **Consequence** | **Reference allele** | **Variant allele** | **Gene** | **Description** | **Validation^b^** | **Segregation^c^** | **Control population^d^** | **MAF^e^ 1000G** |
| --- | --- | --- | --- | --- | --- | --- | --- | --- | --- | --- | --- |
| **49** | 16 | 31326368 | stopgain SNV | C | T | ITGAD | Integrin, alpha D Source:HGNC Symbol;Acc:6146 | Positive | 3/6 | NV | 0.0005 |
|  | 7 | 16468727 | frameshift deletion | A | - | **SOSTDC1** | Sclerostin domain containing 1 Source:HGNC Symbol;Acc:21748 | Positive | 5/6 | 5/734 (0.68%) | N/A |
|  | 7 | 151774352 | frameshift deletion | AG | - | CCT8L1P | Chaperonin containing TCP1, subunit 8 theta-like 1 Source:HGNC Symbol;Acc:32153 | Positive | 3/6 | NV | N/A |
| **694** | 14 | 44737671 | stopgain SNV | C | T | **FANCM** | Fanconi anemia, complementation group M Source:HGNC Symbol;Acc:23168 | Positive | N/A | 5/3896 (0.13%) | 0.0014 |
|  | 5 | 137448086 | exonic;splicing | G | T | **WNT8A** | Wingless-type MMTV integration site family, member 8A Source:HGNC Symbol;Acc:12788 | Positive | N/A | 0/2193 (0%) | N/A |
|  | 1 | 157299120 | exonic;splicing | A | - | AIM2 | Absent in melanoma 2 Source:HGNC Symbol;Acc:357 | Negative | - | - | N/A |
|  | 5 | 11034884 | splicing | - | AAAT | CTNND2 | Catenin cadherin-associated protein, delta 2 neural plakophilin-related arm-repeat protein Source:HGNC Symbol;Acc:2516 | Positive | N/A | NV | N/A |
|  | X | 153658306 | nonframeshift insertion | - | GAA | DKC1 | Small nucleolar RNA, H/ACA box 56 Source:HGNC Symbol;Acc:32650 | Positive | N/A | NV | N/A |
|  | 12 | 64990545 | frameshift deletion | A | - | HELB | Helicase DNA B Source:HGNC Symbol;Acc:17196 | Positive | N/A | NV | N/A |
| **F2887** | 4 | 68249360 | splicing | T | G | UBA6 | ubiquitin-like modifier activating enzyme 6 Source:HGNC Symbol;Acc:25581 | Negative | - | - | N/A |
|  | 12 | 102861703 | frameshift insertion | - | CC | **HSP90B1** | heat shock protein 90kDa beta Grp94, member 1 Source:HGNC Symbol;Acc:12028 | Negative | - | - | N/A |
| **F3311** | 17 | 7791701 | nonsynonymous SNV | A | G | **CNTROB** | centrobin, centrosomal BRCA2 interacting protein Source:HGNC Symbol;Acc:29616 | Positive | 5/8 | 2/192 (1.04%) | N/A |
|  | 12 | 102865327 | frameshift deletion | GAA | - | **HSP90B1** | heat shock protein 90kDa beta Grp94, member 1 Source:HGNC Symbol;Acc:12028 | Positive | 5/8 | 9/185 (4.86%) | N/A |
| **531** | 19 | 54156637 | stopgain SNV | C | T | BAX | BCL2-associated X protein Source:HGNC Symbol;Acc:959 | Positive | 1/5 | NV | N/A |
|  | 14 | 44722732 | nonsynonymous SNV | A | T | **FANCM** | Fanconi anemia, complementation group M Source:HGNC Symbol;Acc:23168 | Positive | 1/5 | NV | N/A |
|  | 1 | 13977729 | nonframeshift insertion | - | AGA | PRDM2 | PR domain containing 2, with ZNF domain Source:HGNC Symbol;Acc:9347 | Negative | - | - | N/A |
| **RUL153** | 22 | 27421857 | frameshift deletion | C | - | **CHEK2** | CHK2 checkpoint homolog S. pombe Source:HGNC Symbol;Acc:16627 | Positive | 3/4 | mutation | N/A |
|  | 7 | 86633821 | nonframeshift deletion | GAT | - | DMTF1 | cyclin D binding myb-like transcription factor 1 Source:HGNC Symbol;Acc:14603 | Negative | - | - | N/A |
|  | 5 | 140167162 | frameshift insertion | - | AAGACACC | PCDHA4 | protocadherin alpha 4 Source:HGNC Symbol;Acc:8670 | Negative | - | - | N/A |
|  | 4 | 68249360 | splicing | T | G | UBA6 | ubiquitin-like modifier activating enzyme 6 Source:HGNC Symbol;Acc:25581 | Negative | - | - | N/A |
| **RUL036** | 4 | 1665238 | splicing | G | A | **SLBP** | stem-loop binding protein Source:HGNC Symbol;Acc:10904 | Positive | 3/4 | 0/745 | N/A |
|  | 17 | 24920468 | nonsynonymous SNV | C | G | TP53I13 | tumor protein p53 inducible protein 13 Source:HGNC Symbol;Acc:25102 | Positive | 2/4 | NV | 0.0023 |
|  | 17 | 7789855 | nonsynonymous SNV | G | A | **CNTROB** | centrobin, centrosomal BRCA2 interacting protein Source:HGNC Symbol;Acc:29616 | Positive | 3/4 | 0/748 | N/A |
|  | 1 | 244565393 | frameshift deletion | G | - | SMYD3 | SET and MYND domain containing 3 Source:HGNC Symbol;Acc:15513 | Positive | 2/4 | NV | N/A |
|  | 1 | 109339789 | nonframeshift deletion | CTC | - | WDR47 | WD repeat domain 47 Source:HGNC Symbol;Acc:29141 | Positive | 2/4 | NV | N/A |

List of 25 potential protein truncating or splicing variants, indels and variants of unknown significance in genes shared by different families. In bold, selected variants.

^a^ Position according to the coordinate system (HG18).

^b^ Sanger sequencing validation.

^c^ Results of segregation studies marking positive individuals out of the total number available for validation.

^d^ Control population studies were performed in variants segregating in over 50% of the available individuals for validation. Figures represent the number of positive cases out of the total number tested, as well as the percentage of positive cases in the population.

^e^ Minor Allele Frequency reported in 1000 Genomes Project (May 2011 release).

N/A = not available.

NV= non validated.
