# Supplementary material for: Whole Exome Sequencing Suggests Much of Non-BRCA1/BRCA2 Familial Breast Cancer Is Due to Moderate and Low Penetrance Susceptibility Alleles
Source: PLoS One. 2013 Feb 8;8(2):e55681. doi: 10.1371/journal.pone.0055681 (PMC3568132; doi:10.1371/journal.pone.0055681)
Supplement: Table S4 — List of Variants Analyzed by Open Array Case-control Design and Results. List of variants analyzed by Open Array case-control design. In bold, selected candidates. aChromosome where the variant was mapped. bPosition according to the coordinate system (HG18). cVariant consequence: NS = non-synonymous variant UTR3 = 3′ untranslated region variant. dFisher’s Exact Test P value. eOdds Ratio. f95% confidence interval for the Odds Ratio. gMinor Allele Frequency reported in 1000 Genomes Project (May 2011 release). N/A = not available. (DOC) [file pone.0055681.s006.doc]

**Table S4. List of Variants Analyzed by Open Array Case-control Design and Results.**

| **Family** | **Chr^a^** | **Position^b^** | **Consequence^c^** | **Reference allele** | **Variant allele** | **Gene** | **Description** | **Allele frequency cases** | **Allele frequency controls** | **P value^d^** | **OR^e^** | **95% CI^f^** | **MAF^g^ 1000G** |
| --- | --- | --- | --- | --- | --- | --- | --- | --- | --- | --- | --- | --- | --- |
| **RUL036** | 16 | 278080 | UTR3 | G | A | **AXIN1** | axin 1 [Source:HGNC Symbol;Acc:903] | 0.0007443 | 0.0001967 | 0.3758 | 3.786 | 0.423-33.89 | N/A |
|  | 17 | 20872549 | NS | G | A | USP22 | ubiquitin specific peptidase 22 [Source:HGNC Symbol;Acc:12621] | 0.0001858 | 0.0001968 | 1 | 0.9442 | 0.05905-15.1 | N/A |
|  | 17 | 8108576 | NS | G | A | PFAS | phosphoribosylformylglycinamidine synthase [Source:HGNC Symbol;Acc:8863] | 0.001863 | 0.001775 | 1 | 1.05 | 0.4261-2.585 | 0.0036 |
|  | 18 | 6940863 | NS | G | A | **LAMA1** | laminin, alpha 1 [Source:HGNC Symbol;Acc:6481] | 0.0003723 | 0 | 1 | N/A | N/A | N/A |
|  | 22 | 31584009 | NS | G | A | **TIMP3** | TIMP metallopeptidase inhibitor 3 [Source:HGNC Symbol;Acc:11822] | 0.0001859 | 0 | 1 | N/A | N/A | N/A |
|  | 7 | 99465440 | NS | C | G | ZKSCAN1 | zinc finger with KRAB and SCAN domains 1 [Source:HGNC Symbol;Acc:13101] | 0.01141 | 0.009657 | 0.39 | 1.184 | 0.8111-1.727 | 0.00491 |
| **531** | 12 | 122378393 | exonic; splicing | T | C | SBNO1 | strawberry notch homolog 1 (Drosophila) [Source:HGNC Symbol;Acc:22973] | 0.000188 | 0.0001977 | 1 | 0.9507 | 0.05945-15.2 | N/A |
|  | 11 | 118749066 | NS | G | A | USP2 | ubiquitin specific peptidase 2 [Source:HGNC Symbol;Acc:12618] | 0.0005382 | 0.000578 | 1 | 0.9311 | 0.1878-4.615 | N/A |
|  | 6 | 34947441 | NS | A | G | UHRF1BP1 | UHRF1 binding protein 1 [Source:HGNC Symbol;Acc:21216] | 0.01433 | 0.0126 | 0.4975 | 1.139 | 0.8157-1.59 | 0.00424 |
|  | 2 | 241714715 | NS | C | T | PASK | PAS domain containing serine/threonine kinase [Source:HGNC Symbol;Acc:17270] | 0.002973 | 0.001772 | 0.2339 | 1.679 | 0.7415-3.804 | 0.00144 |
|  | 1 | 170900118 | NS | A | G | **FASLG** | Fas ligand (TNF superfamily, member 6) [Source:HGNC Symbol;Acc:11936] | 0.0007452 | 0.0001969 | 0.3758 | 3.786 | 0.423-33.88 | N/A |
|  | 14 | 19939034 | NS | C | T | TEP1 | telomerase-associated protein 1 [Source:HGNC Symbol;Acc:11726] | 0.00279 | 0.002164 | 0.5607 | 1.29 | 0.5921-2.812 | 0.00281 |
|  | 1 | 43858431 | NS | C | A | **PTPRF** | protein tyrosine phosphatase, receptor type, F [Source:HGNC Symbol;Acc:9670] | 0.00186 | 0.0007877 | 0.1818 | 2.364 | 0.7409-7.542 | N/A |
|  | 3 | 62164191 | NS | C | A | PTPRG | protein tyrosine phosphatase, receptor type, G [Source:HGNC Symbol;Acc:9671] | 0.0009297 | 0.0009839 | 1 | 0.9449 | 0.2734-3,266 | N/A |
|  | 3 | 69199787 | NS | C | T | **UBA3** | ubiquitin-like modifier activating enzyme 3 [Source:HGNC Symbol;Acc:12470] | 0.000186 | 0 | 1 | N/A | N/A | 0.00051 |
|  | 5 | 80772205 | NS | A | G | SSBP2 | single-stranded DNA binding protein 2 [Source:HGNC Symbol;Acc:15831] | 0.002697 | 0.003083 | 0.7229 | 0.8745 | 0.4319-1.771 | N/A |
| **694** | 14 | 92347652 | NS | G | A | GOLGA5 | golgin A5 [Source:HGNC Symbol;Acc:4428] | 0.005226 | 0.005523 | 0.8937 | 0.946 | 0.5595-1.599 | 0.00234 |
|  | 7 | 24693745 | NS | G | T | MPP6 | membrane protein, palmitoylated 6 (MAGUK p55 subfamily member 6) [Source:HGNC Symbol;Acc:18167] | 0.002914 | 0.002326 | 0.5763 | 1.254 | 0.5926-2.653 | N/A |
|  | 9 | 116707958 | NS | C | T | **TNFSF8** | tumor necrosis factor (ligand) superfamily, member 8 [Source:HGNC Symbol;Acc:11938] | 0 | 0 | 1 | N/A | N/A | N/A |
|  | 3 | 132430138 | NS | G | T | NEK11 | NIMA (never in mitosis gene a)- related kinase 11 [Source:HGNC Symbol;Acc:18593] | 0.0001858 | 0.0001965 | 1 | 0.9454 | 0.05912-15.12 | N/A |
|  | 9 | 127361802 | NS | G | A | **MAPKAP1** | mitogen-activated protein kinase associated protein 1 [Source:HGNC Symbol;Acc:18752] | 0.004278 | 0.001966 | 0.03709 | 2.181 | 1.037-4.587 | 0.00372 |
|  | 16 | 79617698 | NS | C | T | CENPN | centromere protein N [Source:HGNC Symbol;Acc:30873] | 0.01229 | 0.01022 | 0.3545 | 1.204 | 0.8355-1.735 | 0.00096 |
|  | 1 | 45569726 | NS | C | T | MUTYH | mutY homolog (E. coli) [Source:HGNC Symbol;Acc:7527] | 0.0007435 | 0.001968 | 0.1097 | 0.3774 | 0.1183-1.204 | 0.001 |
| **49** | 7 | 16868210 | splicing | G | A | AGR3 | anterior gradient 3 homolog (Xenopus laevis) [Source:HGNC Symbol;Acc:24167] | 0.0009314 | 0.001969 | 0.1996 | 0.4727 | 0.1615-1.384 | 0.0042 |
|  | 19 | 60173206 | NS | C | T | NLRP2 | NLR family, pyrin domain containing 2 [Source:HGNC Symbol;Acc:22948] | 0.004092 | 0.003353 | 0.6309 | 1.221 | 0.6478-2.303 | 0.00642 |
|  | 19 | 60185933 | NS | T | G | NLRP2 | NLR family, pyrin domain containing 2 [Source:HGNC Symbol;Acc:22948] | 0.009848 | 0.0114 | 0.4466 | 0.8622 | 0.5929-1.254 | N/A |
|  | 4 | 35802963 | NS | G | T | ARAP2 | ArfGAP with RhoGAP domain, ankyrin repeat and PH domain 2 [Source:HGNC Symbol;Acc:16924] | 0.002786 | 0.002162 | 0.5608 | 1.289 | 0.5917-2.81 | 0.00047 |
|  | 14 | 22486748 | NS | G | C | **HAUS4** | HAUS augmin-like complex, subunit 4 [Source:HGNC Symbol;Acc:20163] | 0 | 0 | 1 | N/A | N/A | N/A |
|  | 11 | 47264572 | NS | G | A | MADD | MAP-kinase activating death domain [Source:HGNC Symbol;Acc:6766] | 0.0003716 | 0.0005901 | 0.6791 | 0.6296 | 0.1052-3.77 | 0.00049 |
|  | 8 | 22484632 | NS | C | T | **SORBS3** | sorbin and SH3 domain containing 3 [Source:HGNC Symbol;Acc:30907] | 0.0009342 | 0.0003943 | 0.4542 | 2.37 | 0.4597-12.22 | N/A |
|  | 20 | 47135310 | NS | A | G | **CSE1L** | CSE1 chromosome segregation 1-like (yeast) [Source:HGNC Symbol;Acc:2431] | 0 | 0 | 1 | N/A | N/A | N/A |
|  | 20 | 47278712 | NS | C | T | DDX27 | DEAD (Asp-Glu-Ala-Asp) box polypeptide 27 [Source:HGNC Symbol;Acc:15837] | 0.0001858 | 0.0001966 | 1 | 0.945 | 0.05909-15.11 | N/A |
|  | 17 | 45807606 | NS | G | C | EME1 | essential meiotic endonuclease 1 homolog 1 (S. pombe) [Source:HGNC Symbol;Acc:24965] | 0.001115 | 0.0005896 | 0.5092 | 1.892 | 0.4729-7.568 | 0.00091 |
|  | 3 | 142781912 | NS | A | G | RASA2 | RAS p21 protein activator 2 [Source:HGNC Symbol;Acc:9872] | 0.006734 | 0.007516 | 0.6428 | 0.8953 | 0.5666-1.415 | 0.00284 |
|  | 5 | 37043232 | NS | C | G | NIPBL | Nipped-B homolog (Drosophila) [Source:HGNC Symbol;Acc:28862] | 0.000561 | 0.001377 | 0.2158 | 0.4069 | 0.1052-1.574 | N/A |
|  | 4 | 74666436 | NS | C | T | **RASSF6** | Ras association (RalGDS/AF-6) domain family member 6 [Source:HGNC Symbol;Acc:20796] | 0.0007435 | 0.0001965 | 0.3759 | 3.785 | 0.4229-33.88 | N/A |
|  | 2 | 204062554 | NS | G | A | RAPH1 | Ras association (RalGDS/AF-6) and pleckstrin homology domains 1 [Source:HGNC Symbol;Acc:14436] | 0.002883 | 0.002124 | 0.4489 | 1.359 | 0.6299-2.93 | 0.00187 |
|  | 12 | 124075618 | NS | G | A | BRI3PB | - | 0.0003729 | 0.001381 | 0.1005 | 0.2698 | 0.05602-1.299 | N/A |
|  | 9 | 90806008 | NS | G | A | **S1PR3** | sphingosine-1-phosphate receptor 3 [Source:HGNC Symbol;Acc:3167] | 0.000186 | 0 | 1 | N/A | N/A | N/A |

List of variants analyzed by Open Array case-control design. In bold, selected candidates.

^a^ Chromosome where the variant was mapped.

^b^ Position according to the coordinate system (HG18).

^c^ Variant consequence: NS = non-synonymous variant UTR3 = 3' untranslated region variant.

^d^ Fisher's Exact Test P value.

^e^ Odds Ratio.

^f^ 95% confidence interval for the Odds Ratio.

^g^ Minor Allele Frequency reported in 1000 Genomes Project (May 2011 release).

N/A = not available.
