# Supplementary material for: Whole Exome Sequencing Suggests Much of Non-BRCA1/BRCA2 Familial Breast Cancer Is Due to Moderate and Low Penetrance Susceptibility Alleles
Source: PLoS One. 2013 Feb 8;8(2):e55681. doi: 10.1371/journal.pone.0055681 (PMC3568132; doi:10.1371/journal.pone.0055681)
Supplement: Table S5 — Candidate Variants II. Open Array Variants with an OR higher than 2 or absent in control population. In bold, selected candidates. aPosition according to the coordinate system (HG18). bVariant consequence. UTR3 = variant is located at the 3′ untranslated region. NS = non-synonymous variant. cSanger sequencing validation. dResults of segregation studies stating the number of positive individuals out of the total number available for validation in the family. eSHIFT/Polyphen predictions: D = Damaging PD = Probably damaging T = Tolerated. (DOC) [file pone.0055681.s007.doc]

**Table S5. Candidate Variants II.**

| **Family** | **Chromosome** | **Position^a^** | **Consequence^b^** | **Reference allele** | **Variant allele** | **Gene** | **Description** | **Validation^c^** | **Segregation^d^** | **Prediction SIFT/PolyPhen^e^** |
| --- | --- | --- | --- | --- | --- | --- | --- | --- | --- | --- |
| **RUL036** | 16 | 278080 | UTR3 | G | A | **AXIN1** | axin 1 [Source:HGNC Symbol;Acc:903] | Positive | 4/5 | - |
|  | 18 | 6940863 | NS | G | A | LAMA1 | laminin, alpha 1 [Source:HGNC Symbol;Acc:6481] | Positive | 2/5 | T/D |
|  | 22 | 31584009 | NS | G | A | **TIMP3** | TIMP metallopeptidase inhibitor 3 [Source:HGNC Symbol;Acc:11822] | Positive | 3/5 | T/D |
| **531** | 1 | 170900118 | NS | A | G | FASLG | Fas ligand (TNF superfamily, member 6) [Source:HGNC Symbol;Acc:11936] | Positive | 2/5 | D/D |
|  | 1 | 43858431 | NS | C | A | **PTPRF** | protein tyrosine phosphatase, receptor type, F [Source:HGNC Symbol;Acc:9670] | Positive | 5/5 | D/D |
|  | 3 | 69199787 | NS | C | T | **UBA3** | ubiquitin-like modifier activating enzyme 3 [Source:HGNC Symbol;Acc:12470] | Positive | 3/5 | D/D |
| **694** | 9 | 127361802 | NS | G | A | **MAPKAP1** | mitogen-activated protein kinase associated protein 1 [Source:HGNC Symbol;Acc:18752] | Positive | - | T/D |
|  | 9 | 116707958 | NS | C | T | **TNFSF8** | tumor necrosis factor (ligand) superfamily, member 8provided by HGNC | Positive | - | D/D |
| **49** | 14 | 22486748 | NS | G | C | HAUS4 | HAUS augmin-like complex, subunit 4 [Source:HGNC Symbol;Acc:20163] | Positive | 1/6 | T/PD |
|  | 8 | 22484632 | NS | C | T | SORBS3 | sorbin and SH3 domain containing 3 [Source:HGNC Symbol;Acc:30907] | Negative | - | D/D |
|  | 20 | 47135310 | NS | A | G | CSE1L | CSE1 chromosome segregation 1-like (yeast) [Source:HGNC Symbol;Acc:2431] | Negative | - | D/D |
|  | 4 | 74666436 | NS | C | T | RASSF6 | Ras association (RalGDS/AF-6) domain family member 6 [Source:HGNC Symbol;Acc:20796] | Positive | 1/6 | D/D |
|  | 9 | 90806008 | NS | G | A | **S1PR3** | sphingosine-1-phosphate receptor 3 [Source:HGNC Symbol;Acc:3167] | Positive | 3/6 | - |

Open Array Variants with an OR higher than 2 or not present in control population. In bold, selected candidates.

^a^ Position according to the coordinate system (HG18).

^b^ Variant consequence. UTR3 = variant is located at the 3' untranslated region. NS = non-synonymous variant.

^c^ Sanger sequencing validation.

^d^ Results of segregation studies stating the number of positive individuals out of the total number available for validation in the family.

^e^ SHIFT/Polyphen predictions: D = Damaging PD = Probably damaging T = Tolerated.
